# Supplementary material for: Aberrant Mitochondrial Morphology and Function in the BTBR Mouse Model of Autism Is Improved by Two Weeks of Ketogenic Diet
Source: Int J Mol Sci. 2020 May 5;21(9):3266. doi: 10.3390/ijms21093266 (PMC7246481; doi:10.3390/ijms21093266)
Supplement: Supplementary file 1 [file ijms-21-03266-s001.pdf]

International Journal of *Molecular Sciences*

MDPI

Article

Aberrant Mitochondrial Morphology and Function in the BTBR Mouse Model of Autism Is Improved by Two Weeks of Ketogenic Diet

Younghee Ahn [1], Rasha Sabouny [2,†], Bianca R. Villa [1,†], Nellie C. Yee [1], Richelle Mychasiuk [3,4], Golam M. Uddin [2], Jong M. Rho [1] and Timothy E. Shutt [2,*]

[1] Departments of Pediatrics, Clinical Neurosciences, Physiology & Pharmacology, Alberta Children's Hospital Research Institute, Hotchkiss Brain Institute, Cumming School of Medicine, University of Calgary, Calgary, AB, T2N 4N1, Canada; yahn@ucalgary.ca (Y.A.); bianca.villa1@ucalgary.ca (B.R.V.); ncyee@ucalgary.ca (N.C.Y.); jrho@health.ucsd.edu (J.M.R.)

[2] Departments of Medical Genetics and Biochemistry & Molecular Biology, Alberta Children's Hospital Research Institute, Cumming School of Medicine, Hotchkiss Brain Institute, University of Calgary, Calgary, AB, T2N 4N1, Canada; Rasha.Sabouny@ucalgary.ca (R.S.); golammezbah.uddin@ucalgary.ca (G.M.U.)

[3] Department of Psychology, Alberta Children's Hospital Research Institute, Hotchkiss Brain Institute, University of Calgary, Calgary, AB, T2N 4N1, Canada; richelle.mychasiuk@monash.edu

[4] Department of Neuroscience, Monash University, Melbourne, VIC 3004, Australia; richelle.mychasiuk@monash.edu

† These authors contributed equally to this work.

* Correspondence: timothy.shutt@ucalgary.ca; Tel.: +1-403-220-6114

Received: 24 March 2020; Accepted: 27 April 2020; Published: date

Supplementary Materials

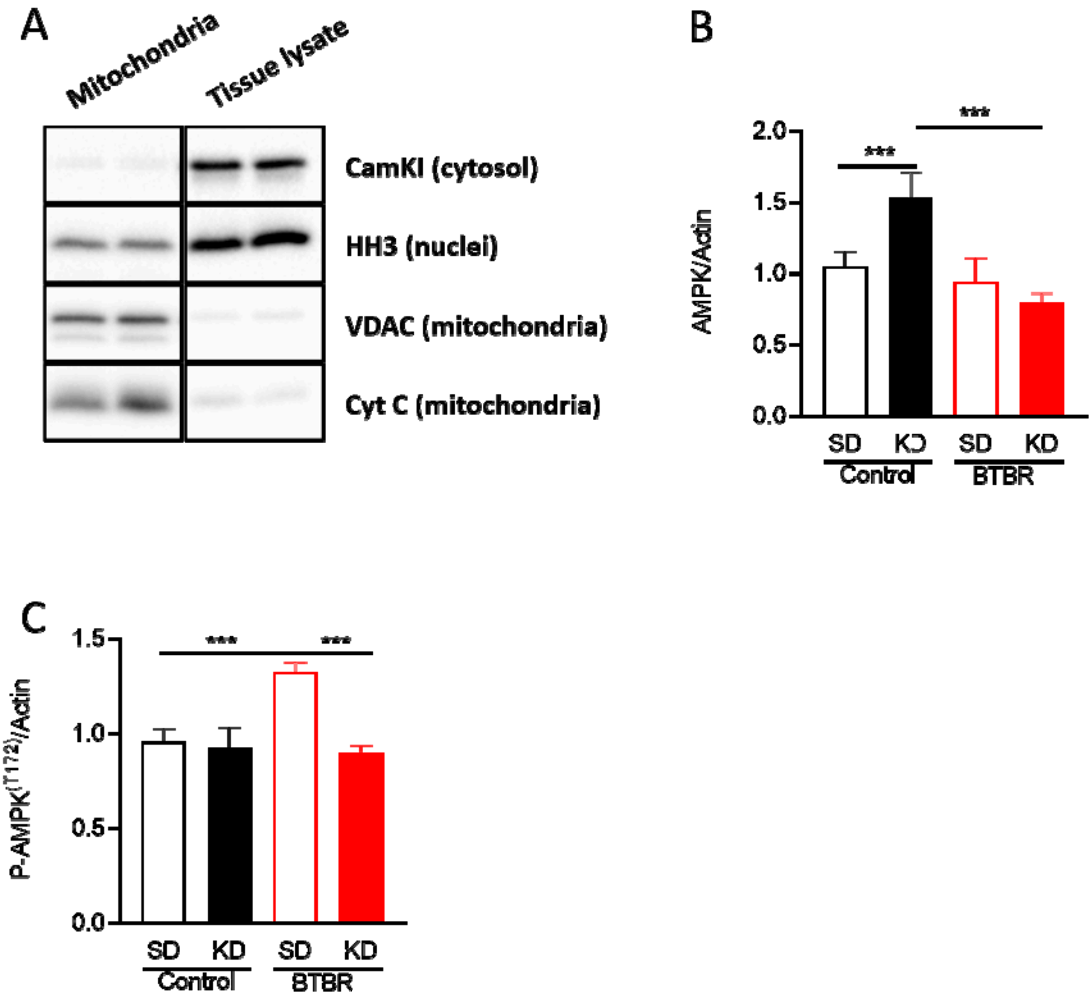

Supplementary Figure 1. Purity of mitochondrial fraction. (**A**) Isolated mitochondria and tissue lysate were analyzed to check for contamination. Western blot with antibodies for characteristic protein of mitochondria (VDAC and Cytochrome C) and cytosol (CamKI). The mitochondrial fraction was free of cytoplasmic contaminants such as CamKI (absent by western blot). (**B**) Densitometry analysis of AMPK and (**C**) P-AMPK $^{Thr\ 172}$ to loading control; (G) Representative blots for the densitometry analysis in figure 2G. Data are shown as mean±SEM, n = 6 per group. Data were analysed by two-way ANOVA. The significant difference between groups reveals by the post-hoc analysis, are presented as: *** P<0.001.

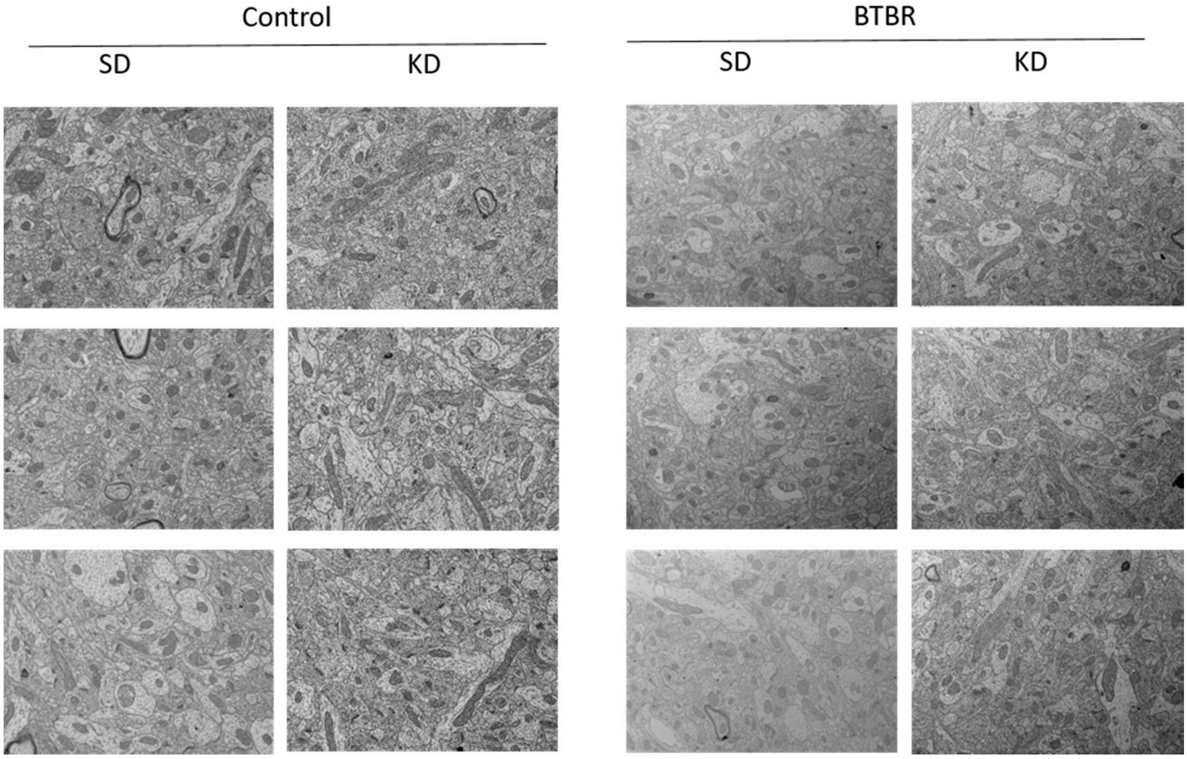

Supplementary Figure 2. Transmission electron microscopy (TEM), 3 representative TEM raw images from each group that was used in quantification. Scale bars represents 500nm.

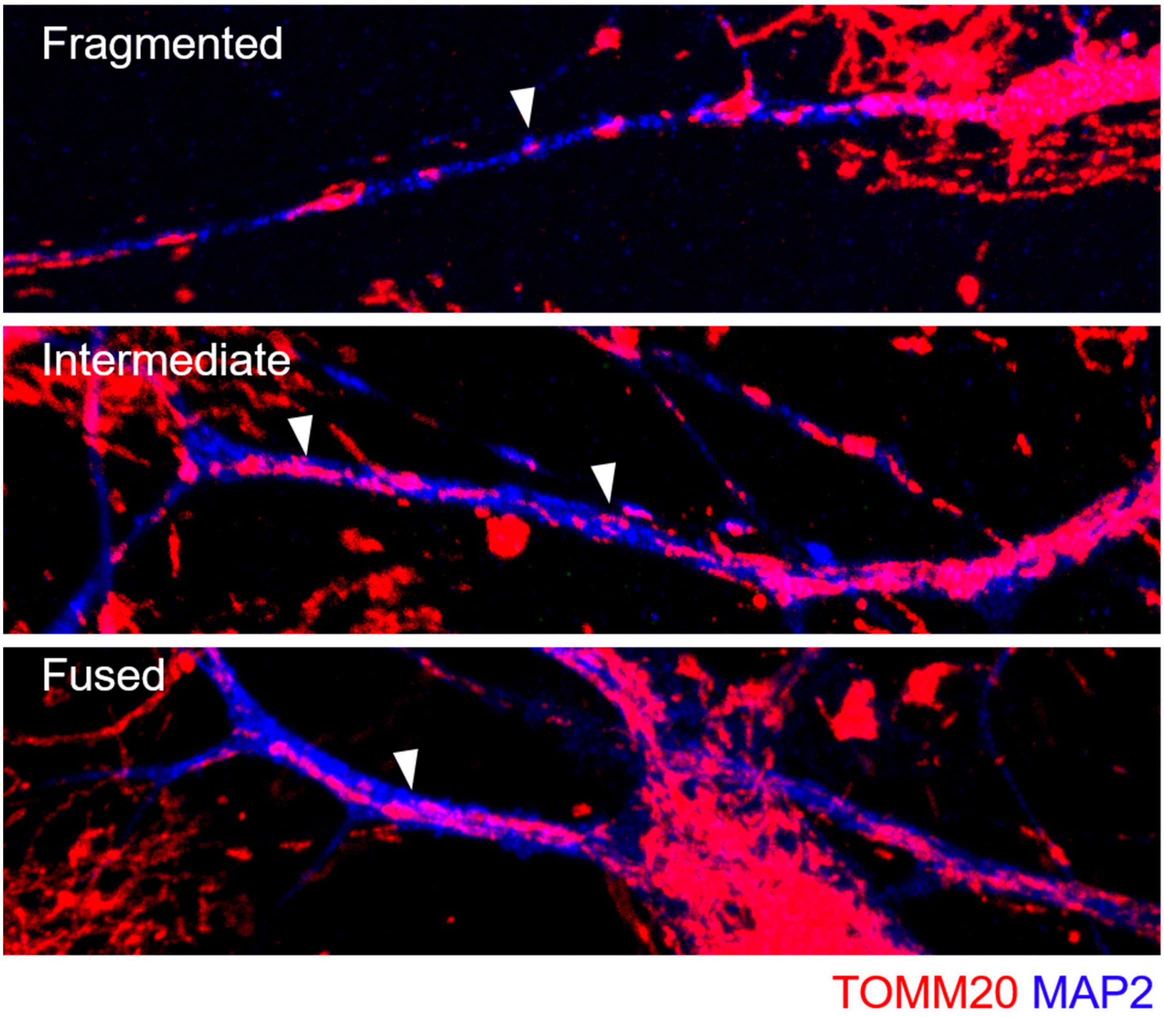

Supplementary Figure 3. Representative confocal images of mitochondrial networks in axonal neurites used as a reference to classify mitochondrial networks in primary neuronal cortical cultures from control and BTBR mice. Quantification of mitochondrial morphology was performed manually by examining at least 50 cells per condition and categorizing them into one of the following classes of mitochondrial network morphology: fragmented (small round puncta), intermediate (a mixture of tubulated networks and small puncta) or fused (primarily elongated tubules). The analyses were performed on 3 independent replicates for each condition.
